# Whole Genome Sequencing and Comparative Genomics of the Emerging Pathogen *Burkholderia pseudomallei* Isolated from Two Travel-Related Infections in Hungary

**DOI:** 10.3390/pathogens14111108

**Published:** 2025-10-31

**Authors:** Judit Henczkó, Ákos Tóth, Márta Knausz, Béla Gartner, Ákos Reményi, Edit Bíró, Erzsébet Létay, László Rókusz, Szilárd Tóth, Bernadett Pályi, Tünde Mag, Tímea Erdősi, Nóra Deézsi-Magyar, Zsuzsanna Molnár, Zoltán Kis

**Affiliations:** 1 Department of National Biosafety Laboratory, National Center for Public Health and Pharmacy, H-1097 Budapest, Hungary; 2 School of PhD Studies, Semmelweis University, H-1097 Budapest, Hungary; 3 Department of Bacteriology, National Center for Public Health and Pharmacy, H-1097 Budapest, Hungary; 4 Microbiology Laboratory, Aladar Petz University Teaching Hospital, H-1097 Győr, Hungary; 5 Central Department of Anesthesiology and Intensive Care, Aladar Petz University Teaching Hospital, H-1097 Győr, Hungary; 6 Department of Otorhinolaryngology, Head and Neck Surgery, North-Pest Central Hospital—Military Hospital, H-1097 Budapest, Hungary; 7 Department of Clinical Microbiology, North-Pest Central Hospital—Military Hospital, H-1097 Budapest, Hungary; 8 Department of Internal Medicine, North-Pest Central Hospital—Military Hospital, H-1097 Budapest, Hungary; 9 Communicable Diseases and Immunization Unit, National Center for Public Health and Pharmacy, H-1097 Budapest, Hungary; 10 Institute of Medical Microbiology, Faculty of Medicine, Semmelweis University, H-1097 Budapest, Hungary

**Keywords:** *Burkholderia pseudomallei*, melioidosis, whole genome sequencing (WGS), core genome MLST (cgMLST), antimicrobial resistance, virulence factors, neuromelioidosis, Europe, emerging infectious diseases, climate change

## Abstract

Background: *Burkholderia pseudomallei*, the causative agent of melioidosis, is a neglected tropical pathogen that has been increasingly encountered in Europe through travel-related infections. Clinical manifestations range from localized abscesses to life-threatening sepsis, posing diagnostic challenges in non-endemic regions. Methods: We report two travel-associated melioidosis cases confirmed in Hungary between 2008 and 2024. Whole-genome sequencing (WGS), multilocus sequence typing (MLST), and core-genome MLST (cgMLST) were performed for molecular characterization. In parallel, a systematic review of travel-related melioidosis cases reported in Europe (1980–2025) was conducted according to PRISMA 2020 guidelines. Data were retrieved from PubMed, Scopus, Google Scholar, and the PubMLST database. Results: In silico MLST identified two distinct sequence types (STs): a novel ST1643, and ST1051, previously reported in Asia and Australia. Both isolates clustered within the Asian clade, confirming an imported origin. Virulence profiling revealed major determinants, including the Yersinia-like fimbriae (YLF) cluster, fhaB3, and ITS type C. The ST1643 isolate carried the *bim*A_Bm_ variant and multiple resistance genes (*bla*OXA-57, *bla*PenI, and *amr*AB efflux system), while ST1051 harbored *bla*OXA-59. The literature review identified 82 studies encompassing 195 European cases, most originating from Southeast Asia, with pneumonia, followed by septic form and abscess as the predominant presentation. We found only eight neuromelioidosis cases in Europe. Conclusions: This study represents the first report of neuromelioidosis in Hungary, and the first global description of ST1643. Combined genomic and epidemiological data highlight the need for improved clinical awareness, genomic surveillance, and diagnostic preparedness in non-endemic regions, as global travel and climate change expand the distribution of melioidosis.

## 1. Introduction

*Melioidosis* (Whitmore’s disease, soil fever) is an emerging infectious disease caused by the environmental bacterium *Burkholderia pseudomallei,* which was first isolated in Burma in 1912 [1,2,3]. It is classified as a risk group 3 (RG3) pathogen and has been designated as a Class B bioterrorism agent by the Centers for Disease Control and Prevention (CDC) [4]. In 2015, the global prevalence of melioidosis was estimated at approximately 165,000 cases, resulting 89,000 deaths [5,6,7]. *B. pseudomallei* is endemic between the latitudes of 20 degrees N and 20 degrees S in Southeast and South Asia, and northern Australia [2,7,8]. The incidence of affected fields has been observed to steadily expand, with 46 countries currently considered endemic. Furthermore, cases have been observed in an additional 33 countries by 2024 [2]. The highest prevalence is found in ‘hyperendemic zones’ in Southeast Asia and northern Australia, with the peak being during the rainy season [9]. Global transportation systems, particularly transport of contaminated products, may facilitate bacterial spread [10]. The *Burkholderia* genus is known for its extreme surviving ability and broad resistance to many disinfectants. It has been widely reported that *B. pseudomallei* can survive for up to two decades in distilled water or in different beverages [11,12,13,14,15]. Melioidosis is recognized as a zoonosis, as a wide range of animals are susceptible to the bacterium, including iguanas, non-human primates, dogs, crocodiles, goats, and sheep, although animals, as well as humans, are usually dead-end hosts [8,16,17,18]. The most common acquisition of human infections is via exposure to various contaminated environmental materials by inhalation, ingestion, or direct contact with abraded skin [9]. More than 99% of the fatal cases occur predominantly among agricultural workers in the endemic areas [16]. Person-to-person transmission, including sexual transmission or vertical transmission through breastfeeding, is rare but possible [19,20,21,22]. The typical incubation period varies from 1 to 21 days with 4 days [23,24]. The infection can potentially remain latent for up to 62 years, or reactivate after months or years, as the bacteria can evade the host’s immune system [25].

The infection can be localized or disseminated, and the most frequent type of melioidosis is pneumonia, but sepsis, multiple abscesses, and other forms such as wound infections, genitourinary infections, enteric infection, even neurological forms can also occur [21,26,27,28,29,30,31]. Differentiation from the clinical presentation of some malignant diseases, autoimmune diseases, tuberculosis, malaria, rickettsiosis, leptospirosis, plague, dengue, or staphylococcal bacteraemia may pose a considerable challenge [32]. Reinfections are not uncommon, as there is no cross-protection between different genotypes. Individual susceptibility to melioidosis is associated with several predisposing health conditions, including, but not limited to, diabetes mellitus, cirrhosis, chronic renal disease, cancer, and alcoholism [33,34]. The mortality rate of melioidosis usually varies from 14 to 50%, depending on the condition of the patient, and the lack of prompt and appropriate therapy and its fulminant septic form is still nearly 100% fatal [21,28,29,30,34,35,36].

*B. pseudomallei* possesses intrinsic resistance to several commonly used antibiotics, including penicillin, first- and second-generation cephalosporin, and macrolide. Consequently, intensive treatment of melioidosis typically relies on ceftazidime or meropenem. Although acquired resistance to these frontline agents remains relatively rare, it can arise through alterations in penicillin-binding proteins, increased β-lactamase production, or modifications to efflux pump activity. Globally, resistance rates vary, with higher prevalence reported for tetracyclines and ciprofloxacin, underscoring the importance of early detection and careful antibiotic selection to ensure effective therapy [37]. Currently, the first-choice therapy is initially the IV administration of ceftazidime or meropenem for 10–14 days, followed by trimethoprim/sulfamethoxazole per-os (or amoxicillin–clavulanate or doxycycline as alternatives) for 3–6 months [38]. The patient could recover without any residual symptoms, but fatal outcomes or relapses can occur despite the adequate therapy [39]. Currently, no effective vaccines are available [40].

Regarding microbiological diagnosis, cultivation of the organism is the gold standard, but it has a low sensitivity, and hence, a low negative predictive value. Consequently, molecular diagnostic methods, including PCR-based, CRISPR-Cas-based or whole genomic-based methods slowly become first line diagnostic methods; however, standardization is still ongoing [41,42].

*B. pseudomallei* possesses one of the largest bacterial genomes (~7.2 Mb), distributed across two chromosomes. It is heavily armed with virulence factors that enable it to survive in extreme environments and cause a wide range of clinical disease presentations [43]. The prognosis of the infection may relate to the presence or absence of the different virulence factors, including capsule, pili, flagella, lipopolysaccharide (LPS), quorum-sensing members, two types of Type 3 secretory systems (T3SS), Type 6 secretory systems (T6SS) and exotoxins [44,45]. Toxin A (ToxA) is a part of T3SS and plays a significant role in the ability to infect and damage host tissues. Moreover, ToxA can enter the host cell and interfere with key cellular processes like cell cycle regulation, actin cytoskeleton dynamics, and apoptosis, often leading to cell death and tissue damage [44,45]. A key virulence factor, the putative autotransporter *Burkholderia* intracellular motility A (BimA), enables *B. pseudomallei* and *B. mallei* to move via actin-based motility. This protein also aids bacterial spread and shields them from autophagy [45,46]. Understanding BimA and its role in *B. pseudomallei* infection is crucial, because it could help identify potential therapeutic targets. By inhibiting BimA, it might be possible to reduce the ability of bacteria to spread in the host, potentially reducing the severity of melioidosis [46].

*B. pseudomallei* belongs to the Type II O-polysaccharide and has three different LPS types: namely, A, B, and B2. The LPS gene can change the phenotype without changing the genotype [47]. LPS type A is commonly found in Asia, LPS type B in Australia and Asia, and LPS type B2 in Australia, as well [48]. The internal transcribed spacer (ITS) typing differentiates B. *pseudomallei* strains based on ITS region variation, providing useful information for epidemiological studies and tracking sources of infections. In the case of *B. pseudomallei*, ITS types are often classified into different categories based on sequence analysis. ITS type A is typically found in isolates from Southeast Asia, especially from Thailand and Malaysia, while ITS type B is often associated with isolates from Australia. ITS type C is frequently observed in isolates from India and other parts of Asia [45]. *B. pseudomallei* possesses three filamentous hemagglutinin genes (*fha*B), of which the *fha*B3 variant has been associated with positive blood cultures and is negatively correlated with localized skin infections, mainly without sepsis [44,45]. *Yersinia*-like fimbriae (YLF) and *Burkholderia thailandensis*-like flagellum and chemotaxis (BTFC) gene clusters are also known to have distinct geographic distributions. The YLF gene cluster has been associated more with clinical isolates than with environmental ones [39,44,45].

While cases among returning travelers are frequent worldwide, *B. pseudomallei* epidemiology in Europe remains poorly understood and likely underreported [35,49]. In Europe, melioidosis is currently not listed as a communicable disease threat, according to the Commission Implementing Decision (EU) 2018/945 of 22 June 2018. Climate change is expected to influence the spread of infectious diseases in Europe by creating environmental conditions that favor bacterial growth and establishment. Understanding *B. pseudomallei*, especially its emergence in Europe, highlights the need for increased surveillance and innovative strategies to manage its threats. The pathogenicity of *B. pseudomallei*, alongside its resilience against common antibiotics, calls for urgent attention, particularly given its historical implications as a potential biological threat, as highlighted by its notorious use during World War II [45,50]. Therefore, establishing robust preventative measures and fostering interdisciplinary research efforts will be crucial in mitigating the risk that *B. pseudomallei* poses to human and animal health in Europe and beyond. However, until now, autochthonic cases in connection with *B. pseudomallei* were not confirmed in Europe [51,52].

## 2. Materials and Methods

### 2.1. B. pseudomallei Isolates

In Hungary, between 2008 and 2024, the National Reference Laboratory for Highly Pathogenic Bacteria at the National Center for Public Health and Pharmacy tested different types of clinical specimens from 100 patients with travel anamnesis from well-known endemic areas. Moreover, 3 bacterial isolates were submitted to the laboratory for confirmation, from which 2 were confirmed as *B. pseudomallei*, and 1 was *Pseudomonas aeruginosa*. All procedures with living cultures were performed under BSL-3 conditions at the National Biosafety Laboratory, National Center for Public Health and Pharmacy (Table 1). The bacterial strain stocks were stored at −80 °C in cryoprotective media for further analysis. For whole genomic sequencing, the strains were grown on a Luria–Bertani (LB) agar plate for 24 h at 37 °C ambient air. The purity of the strains was checked by using Matrix-Assisted Laser Desorption/Ionization Time-of-Flight, MALDI-TOF MS (Bruker Daltonics Inc., Bremen, Germany) after a trifluoroacetic acid (TFA) inactivation method, as described previously [53].

### 2.2. Molecular Methods

DNA isolation was performed from the isolates by using the QIAamp DNA Mini Kit (Qiagen, Hilden, Germany) according to the manufacturer instructions. Multiple qPCRs were performed, targeted to the *orf*2 of the Type III Secretion System (T3SS) [54]. After a quality check by using a Qubit Flex instrument (Thermo Fisher Scientific, Grand Island, NY, USA), the DNA libraries were prepared by using an Illumina DNA Prep kit (Illumina, Inc., San Diego, CA, USA), following the manufacturer instructions without any modifications to the protocol. Pooled libraries were quantified by using the Qubit Flex instrument (Thermo Fisher Scientific, Grand Island, NY, USA). Whole genome sequencing was performed on the Illumina MiSeq platform (Illumina Inc., San Diego, CA, USA), using MiSeq reagent kit version 2, for 300 cycles (Illumina Inc., San Diego), generating 2 × 150 bp paired-end sequencing runs. For bioinformatics analysis, a custom-made pipeline was developed. Briefly, the raw reads were checked with FastQC version 0.12.0, and reads with a Phred score under 20 were discarded [55]. Quality-based filtering and trimming were performed using TRIMMOMATIC version 0.39, and de novo assembly was conducted using Spades version 3.15.3, with the careful option to perform mismatch correction [56,57]. Structural gene prediction and functional annotation was performed with the Bakta version 1.11.3 and RAST server [58,59]. The presence of potential CRISPR-Cas systems was checked using a CRISPRCasFinder version 2.0.3 [60]. Virulence-associated gene analysis and annotation was conducted by using a custom-made database, originally downloaded from the Virulence Factor Database (VFDB) downloaded on 30 September 2025, and the analyses were performed with the MyDBFinder 2.0 and Ridom SeqSphere+ version 8.0 tools (www.ridom.de, accessed on 30 September 2025) [36,61]. Assembled genomes were analyzed for the identification of resistance genes using the Comprehensive Antibiotic Resistance Database (CARD) and the ResFinder database. Only resistance genes exhibiting a coverage of >80% and an identity (proportion of exact nucleotide matches) of >75% were accepted [62]. Further typing of the two assembled genomes was performed using a Ridom SeqSphere+ [63] Additionally, 410 *Burkholderia pseudomallei* genomes from the National Center for Biotechnology Information (NCBI) were downloaded (Supplementary Data S1). K96243 was used as a reference genome. The public database for molecular typing (PubMLST) web software was used to determine the in silico MLST sequence types (http://pubmlst.org/bpseudomallei/, accessed on 30 September 2025). The relatedness of *B. pseudomallei* isolates, based on their MLST profiles, was manually verified using the MLST database (http://eburst.mlst.net/). Concatenated sequences for all sequence types (STs) included in this study were downloaded from the MLST website (http://pubmlst.org/bpseudomallei/, accessed on 30 September 2025). A minimum spanning tree, based on cgMLST, was constructed using RIDOM SeqSphere+ (Münster, Germany) and visualized by iTOL version 7.2 [64]. Furthermore, the PubMLST isolate database (n = 7510) was analyzed and compared with the two isolates under investigation, using GrapeTree version 1.52.0 [65]. Visualization and extraction of the two samples’ related information was performed by using GrapeTree.

### 2.3. Update on European Melioidosis Cases

The review followed the Preferred Reporting Items for Systematic Reviews and Meta-Analyses (PRISMA) guidelines for systematic literature reviews.

#### 2.3.1. Search Strategy

A search was conducted for the literature review; the search covered 1980–2025 and the literature was obtained from PubMed, Web of Science, PubMLST, and a web-based direct search for five specific search criteria: “*Burkholderia pseudomallei* in Europe” “melioidosis cases in Europe” and “neuromelioidosis”, and “melioidosis travel related”. Additionally, melioidosis cases were extracted from the pubMLST database [66]. All articles with available online full texts that were published in peer-reviewed journals and country reports until 30 September 2025 were included, with no restrictions for gender, age group, region, or the language in which they were published. The English, German, and French languages were included, but we did not exclude results in other languages either. Citation searching was performed to look for additional articles. The articles were separately screened. Titles and abstracts based on the inclusion/exclusion criteria were checked. Full texts of the articles, or reports, were independently selected according to the eligibility criteria. All articles or reports described clinical data relating to human cases of melioidosis in Europe. Discrepancies in the decision to include the study and data extraction were resolved through consensus and by including a third reviewer.

#### 2.3.2. Eligibility Criteria

Patients of all ages who were diagnosed with melioidosis were screened for eligibility.

#### 2.3.3. Data Extraction and Analysis

Two reviewers extracted data independently, using a standardized form containing study characteristics (author, year of publication) and patient demographics. For each variable, only cases where the detail was explicitly reported as present or absent were included in the denominator, with the number reported as present serving as the numerator. Data on epidemiology (geographic area of presentation, comorbidities), clinical signs and symptoms (neurological findings, etc.), diagnosis (method of diagnosis and specimen type), and clinical outcomes (death, cure with or without residual neurological deficits) were collected. The data were recorded in a Microsoft Excel spreadsheet (Supplementary Data S2).

##### Nucleotide Sequence Accession Numbers

Sequences have been deposited at the Sequence Read Archive (SRA) under the accession of SUB12019197, BioSample: SAMN26814095 and SAMN26814096.

## 3. Results

### 3.1. Whole-Genomic Sequencing and Comparative Genomics Results

The main genomic characteristics of the two Hungarian isolates are summarized in Table 2. Genome sizes were 7.2 Mb (584_2008_OEK) and 7.13 Mb (831_2019_NNK), with GC contents of 67.9% and 68.1%, respectively. Both genomes contained ~7400 predicted coding sequences and 299–300 annotated virulence-associated genes. Differences were noted in the number of tRNA and rRNA genes, but both isolates harbored comparable numbers of non-coding RNAs and other genomic elements.

### 3.2. In Silico Multilocus Sequence Typing

In silico MLST assigned isolate 584_2008_OEK to a novel sequence type, ST1643, carrying a previously undescribed *ndh* allele. This new ST has been deposited in the pubMLST database. Isolate 831_2019_NNK belonged to the previously reported ST1051 (Table 3).

As of June 2025, the pubMLST database contained 7510 isolates, of which only 74 originated from Europe and 58 (78.4%) were linked to human disease, including the two isolates from Hungary. The origin of infection remains unknown for 33 cases (44.6%), while 24 cases (32.4%) were attributed to Asia and 7 (9.4%) to Africa, while 1 (1.4%) was attributed to the Caribbean Islands. Data regarding the molecular analysis of South Asian strains remain limited. ST1051 has seven entries in the database, reported from clinical isolates in Vietnam (n = 2) and India (n = 2), and environmental isolates in Australia (n = 3). The isolate 584_2008_OEK was a single ST (ST1643, ID5254). The isolate 831_2019_NNK was assigned to an existing sequence type, ST1051, which has seven records in the pubMLST database (Table 4).

### 3.3. cgMLST Analysis

**Figure 1:**
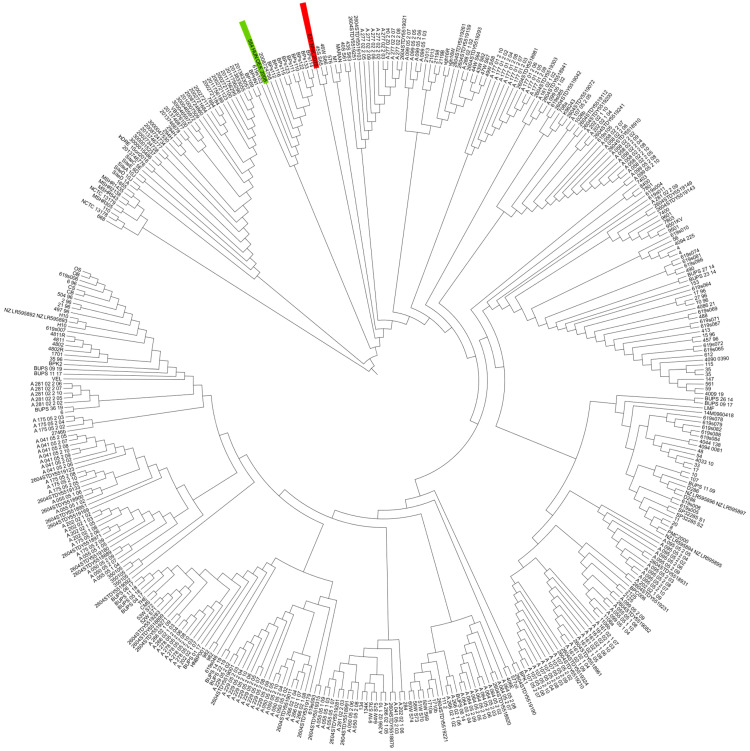
Comparison of Hungarian *B. pseudomallei* strains (n = 2) with global isolates (n = 410). The tree was built based on cgMLST. The NJ-tree shows clustering of global isolates like that of a previously constructed global SNP phylogeny, and provides high resolution for closely related isolates on a global level. The cgMLST-based neighbor-joining tree of 401 global *B. pseudomallei* isolates is constructed by using 4221 target genes. K96243 was used as the reference strain. The red line shows the clade where 831_NNK_2019 was included and the green line shows the clade where 584_OEK_2008 was included.

Comparative cgMLST was performed with 410 global *B. pseudomallei* genomes obtained from NCBI. Both Hungarian isolates showed marked genetic divergence from each other (Figure 1). Isolates 584_2008_OEK and 831_2019_NNK were positioned in distinct clades, consistent with their different sequence types.

**Figure 2:**
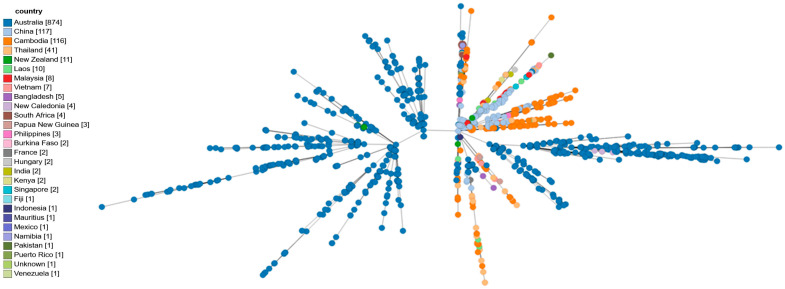
Allelic profiles of 7510 isolates from the PubMLST database. The visualization was performed using GrapeTree. Each node represents a distinct sequence type (ST) and is colored by geographic origin. Hungarian isolates clustered within the Asian lineage, showing close allelic relationships (1–3 loci difference) to sequence types from Southeast Asia, including Thailand, Vietnam, and India. The overall topology demonstrates a clear separation between Asian and Australian clades, while isolates from Africa and the Americas form distant clusters. These findings support the imported origin of Hungarian cases and reflect the global population structure of *B. pseudomallei.* Data source: PubMLST (accessed January 2025).

**Figure 3:**
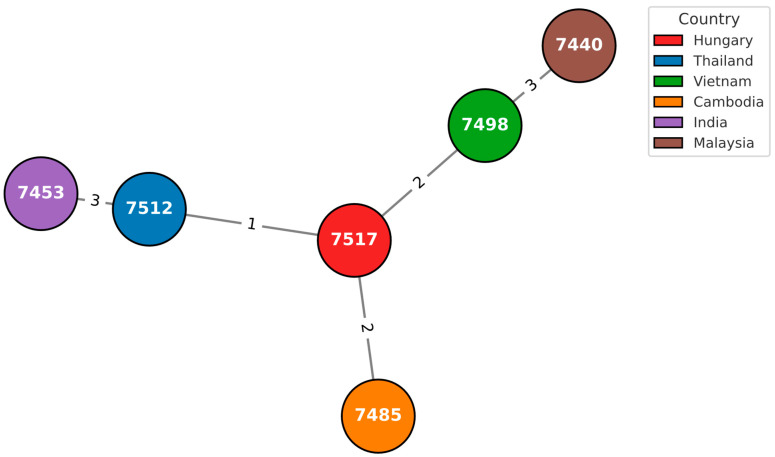
GrapeTree analysis of *Burkholderia pseudomallei* isolates, based on MLST data (PubMLST ID 7517). Each node represents a unique sequence type (ST), while colors correspond to the country of origin. Node ID 7517, representing the Hungarian isolate with the novel sequence type ST1643, is highlighted within the Asian cluster. The black connecting lines indicate the number of allelic differences between isolates, illustrating the genetic relationships among them. The position of ID 7517 shows a limited allelic overlap with neighboring isolates, suggesting an independent lineage within the Asian clade. Nearby nodes, such as ID 7512, ID 7498, and ID 7453, represent reference isolates from Southeast Asia, showing limited allelic overlap with 7517. Numbers displayed on the connecting lines indicate allelic distances, i.e., the number of MLST loci differing between isolates. The position of ID 7517 within the network shows minimal connectivity from larger allelic distances to neighboring nodes, suggesting that ST1643 forms an independent lineage within the Asian clade. Colors correspond to the geographic origin of isolates, as retrieved from the PubMLST database (accessed January 2025). Figure 3 demonstrates the phylogenetic position of the novel sequence type ST1643 (ID 7517). This strain shows limited allelic overlap with known isolates, suggesting an independent lineage within the Asian clade. Its unique ndh 87 allele confirms its status as a previously undescribed genotype, now deposited in the PubMLST database.

**Figure 4:**
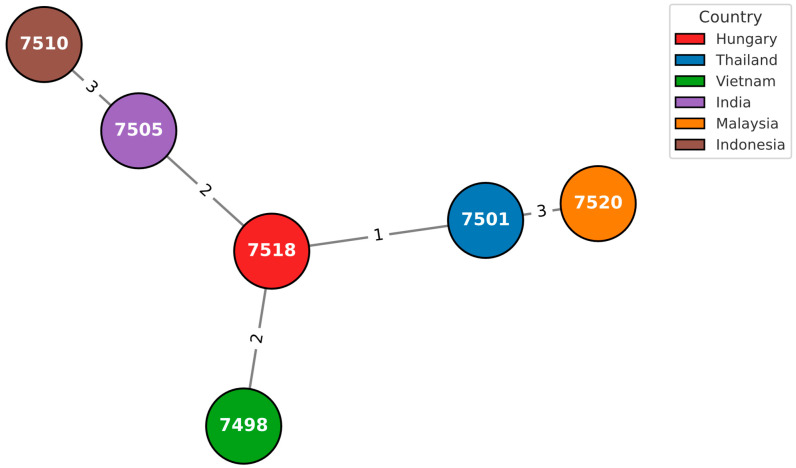
*B. pseudomallei* Grapetree screen on ID 7518, based on the MLST data. Each node represents a distinct isolate labeled with its PubMLST ID, and node colors correspond to the country of origin. The Hungarian isolate (ID 7518, ST1051) occupies the central position within the network and shares short allelic distances (1–3 loci) with Southeast Asian isolates, including Thailand (ID 7501), Vietnam (ID 7498), and India (ID 7505). The connecting lines denote the number of differing MLST loci, reflecting close genetic relatedness to strains circulating in Asia. This pattern supports the imported origin of the Hungarian isolate within the established Asian clade. Colors correspond to the countries listed in the legend. Data source: PubMLST (accessed January 2025).

A total of 7510 isolates were analyzed and compared with the two isolates under investigation using the GrapeTree module on PubMLST. Visualization and extraction of sample-specific information were performed with GrapeTree, enabling detailed comparative analysis of the two isolates within the global dataset (Figure 2, Figure 3 and Figure 4).

### 3.4. Antimicrobial Resistance Genes

Isolate 584_2008_OEK carried *bla*OXA-57, *bla*PenI, and the efflux pump operon *amr*AB, along with the outer membrane protein *omp38*. In contrast, isolate 831_2019_NNK contained only *bla*OXA-59, a gene commonly present in *B. pseudomallei*.

### 3.5. Virulence-Associated Genes

Draft genomes were analyzed to predict the presence of relevant virulence-associated genes. Both strains were found to harbor the most relevant *B. pseudomallei*-associated known virulence factors (Supplementary Data S3).

According to the RAST server, the main virulence-associated subsystems were identified (Supplementary Data S4). Isolate 584_2008_OEK contained 299 virulence-associated genes, while the isolate 831_2019_NNK contained 300 virulence-associated genes. The strain 584_2008_OEK was classified as LPS serotype A, while strain 831_2019_NNK was LPS serotype B. Furthermore, our data revealed that the entire *wcb* operon (BPSL2786 and BPSL2810), which was crucial for LPS biosynthesis, was present in both isolates, without key attenuating mutations [48]. Both strains harbored the Yersinia-like fimbria (YLF) gene cluster, but were negative for the *B. thailandensis*-like flagellum and chemotaxis (BTFC) gene cluster [45]. We also identified the *B. mallei*-like actin polymerization gene (*bim*A), with the *bim*A*_Bm_* variant in strain 584_2008_OEK and the *bim*A*_Bp_* variant in strain 831_2019_NNK. Both strains also tested positive for *fha*B3, and when exhibiting a 622 bp sequence length corresponding to ITS type C, we observed the presence of the *tox*A gene, which encodes for the toxin A protein [45]. The isolate 584_2008/OEK contained *bla*OXA-57 and *bla*PenI β-lactamases in addition to *amr*AB genes, encoding an efflux system [42]. The outer membrane protein Omp38 was also detected. In contrast, isolate 831_2019_NNK contained only the blaOXA-59 gene [43].

### 3.6. Literature Review on Neuromelioidosis Cases in Europe

**Figure 5:**
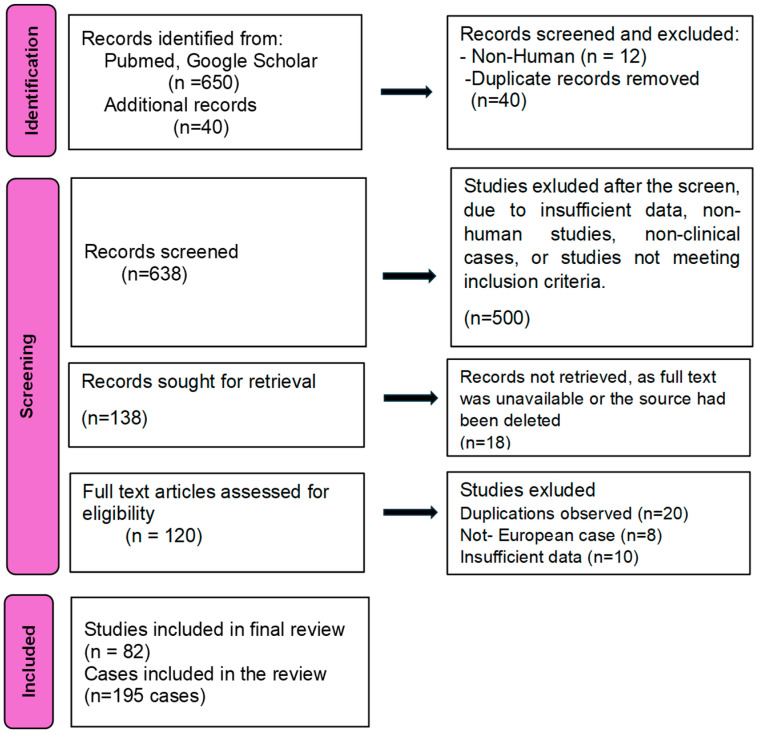
PRISMA flow diagram for selections of melioidosis clinical cases in Europe between 1980 and 2025.

By September 2025, at least around 195 imported melioidosis cases had been documented across Europe between 1980 and 2025 (Figure 5). As of 2019, 184 cases were recorded. After 2019, seven more cases were recorded, until 2024 [8,35,67,68]. Additionally, we found four more cases in 2025 [8,35,69]. A total of 195 cases were recorded between these periods (Table 5) [35,70]. The majority of affected patients were male: (n = 146) 74% of the total cases; median age was 49%. Only one pediatric case was observed. Co-morbidities were poorly captured, according to the absence of surveillance mechanisms in Europe. Respiratory diseases, sepsis, and abscess formation were the most common presenting features. The most cases have been reported in the UK (n = 69), followed by The Netherlands (n = 39), France (n = 24), and Germany (n = 14). The most frequent travel anamnesis was Thailand with 73 cases.

Furthermore, we found only nine neurological cases in Europe, including meningitis, meningoencephalitis, and cerebral abscess; all patients were men between 26 and 58 years old (median age was 50 years), and Thailand was the most frequent travel destination (n = 4), followed by India (n = 1), Cambodia (n = 1), and Sri Lanka (n = 1). One patient visited several countries before the infection, and in one case, the travel anamnesis was not specified in the manuscript (Table 6).

The pulmonary-related melioidosis form was frequent, with a total of 46 cases, followed by the unspecified category and cutaneus/soft tissue infections. The literature discusses only three possible autochthonous occurrences in Europe: one represents Bologna, Italy, where *B. pseudomallei* was reported in tap water (6 out of 85 specimens) in 2000. However, the confirmation of *B. pseudomallei* by any specific laboratory methods has never been reported. Moreover, a soil culture was positive for *B. pseudomallei* in the ‘Jardin des Plantes’ in Paris; this was identified after melioidosis infections, which were connected to a panda imported from China. Only one potential human autochthonous case was reported in Germany, but there is insufficient evidence available at present, because the incubation period of melioidosis can be years, and the patient traveled a lot two years earlier, but detailed information is not available [29].

## 4. Discussion

This study aimed to provide an analysis of whole genome sequencing data from *B. pseudomallei* clinical isolates associated with travel-related infections in Hungary, and also to provide a short update on European cases.

Between 2008 and 2024, two *B. pseudomallei* strains were identified in Hungary from travel-related infections. The first case, in 2008, presented with meningoencephalitis. Neuromelioidosis is rare, with fewer than 100 cases reported worldwide, and it carries a ~25% mortality rate, even with optimal therapy [28,29,32]. Survivors often experience long-term neurological deficits [29,32]. In Europe, delayed recognition may worsen outcomes, as neuromelioidosis can mimic autoimmune neurological syndromes, including an acute disseminated encephalomyelitis (ADEM)-like syndrome, or a Guillain–Barré-like syndrome, or it can present as a spatial sparsii, tomiso. Recognizing the correct etiology is critical because treatments for these conditions are completely different [32]. *B. pseudomallei* therapy is based on prolonged antibiotic therapy, whereas ADEM and Guillain–Barré are immune-mediated, requiring immunosuppressive or immunomodulatory therapy. Misdiagnosis can be fatal, making prompt recognition and correct therapy crucial [28]. In contrast, a neck abscess is a common manifestation of the disease, and belongs to the one of the most frequent forms worldwide. In the literature, we found only eight neuromelioidosis cases in Europe and according to our knowledge, 584_OEK_2008 was the first sequenced meningoencephalitis case in Europe. The absence of pathognomonic features has been a real public health concern in recent years [2,9,16]. *B. pseudomallei* exemplifies the complexities of infectious diseases that transcend geographical boundaries. Delayed diagnosis of travel-related melioidosis in non-endemic regions frequently leads to increased morbidity and mortality. The global incidence of melioidosis has sharply increased, reflecting the growing severity of this worldwide health concern [2,9,16].

The high-level genetic diversity among different strains, particularly the different carrying genes involved in extreme adaptability to different environmental niches, makes it possible to estimate the survival ability in the temperate climate and the real burden of *B. pseudomallei* in Europe [18]. Currently, the environmental risk of *B. pseudomallei* in Hungary is considered low. Global suitability models show that the bacterium predominantly thrives in tropical and subtropical climates, whereas as currently, the climate in Hungary is temperate–continental, conditions generally did not favor its persistence in soil or water. However, climate change and extreme weather events, such as heavy rainfall or flooding, could increase local environmental suitability, particularly in warmer and wetter areas or regions with standing water, like irrigated fields or floodplains. Although the risk remains low, targeted monitoring is advisable among travelers. This includes raising clinical awareness in hospitals and the reference laboratory to ensure prompt identification of potential melioidosis cases, conducting environmental sampling using established international protocols, and maintaining epidemiological surveillance of both human and animal infections, including imported cases. Updating local risk models with regional climate and soil data can further help to identify areas that may become vulnerable in the future [8,16,51,75]. The literature discusses only three possible autochthonous occurrences in Europe; however, there is currently insufficient evidence to conclusively prove the presence of bacteria [29,76].

In *B. pseudomallei*, the currently used sequencing technologies and bioinformatics analysis pipelines often struggle to discriminate properly between different strains. This limitation arises from several factors, including MLST sequence type (ST) homoplasy, sequencing bias, and the lack of dedicated analysis pipelines for ‘chimeric’ or ‘genetically engineered’ microorganisms [41,77]. The in silico MLST analysis of the isolate from our neuromelioidosis case revealed a novel *ndh* (*ndh* 87) allele, which belonged to a new sequence type, ST 1643. ST 1051 showed significant genetic overlap with Australian strains, despite the presence of Yersinia-like fimbriae (YLF), a feature typically associated with Asian strains. This observation aligns with the concept of homoplasy, a common challenge in genomics-based studies of *B. pseudomallei* strains. Homoplasy occurs despite the different geographical origin and virulence features, which can determine the geographical location as well. Our results also highlight the problem that there are currently no reliable genotyping methods available for *B. pseudomallei*, which is obstructing the epidemiological investigations, as well as identification in the case of a potential bioterror event [41,77].

According to our results, we identified almost 300 virulence-associated genes in the genomes, including critical factors that are likely to play an important role in human virulence. Genomic analysis revealed the presence of the Yersinia-like fimbria (YLF) gene cluster in both strains. This cluster, frequently observed in Asian strains and known for its horizontal acquisition, is implicated in host–cell adhesion and biofilm formation, thereby enhancing pathogenicity. In contrast, neither strain possessed the *B. thailandensis*-like flagellum and chemotaxis (BTFC) gene cluster, a genetic element that is characteristic of Australian isolates. The BTFC cluster is recognized for its role in motility and chemotaxis, which contributes to bacterial adaptation across various environmental niches [44,47]. We also identified the *B. mallei*-like actin polymerization gene (*bim*A), with the Bm variant in strain 584_2008_OEK and the Bp variant in strain 831_2019_NNK. The BimA Bm variant is strongly associated with neurological disease, as indicated by several publications [27,28,29,30,60]. BimA plays a critical role in actin-based motility within host cells, facilitating bacterial invasion and dissemination. Furthermore, the BimA Bm is a potential target for CRISPR-Cas-based detection methods in neurologic melioidosis cases. Such cutting-edge methods are particularly important in non-endemic areas, as their limit of detection is significantly lower than the currently available diagnostic approaches [42]. Both strains exhibited an ITS with a 622 bp sequence length, corresponding to ITS type C: a characteristic commonly observed in Asian isolates [41]. Both strains tested positive for *fha*B3. FhaB3 is implicated in the adhesion to host cells and tissue invasion, contributing to the strain’s ability to persist in human and animal hosts. This gene is frequently associated with increased virulence, particularly in strains that cause severe infections, such as pneumonia or sepsis. The presence of *fha*B3 further suggests the potential for these strains to exhibit enhanced pathogenicity in clinical settings [44,45]. The *B. pseudomallei* capsule polysaccharide is a critical virulence factor that is essential for immune evasion. Our data revealed that the entire *wcb* operon (BPSL2786 and BPSL2810) was present in both isolates, with no key mutations identified that could potentially attenuate the strains [48]. The capsule plays a significant role in resisting phagocytosis by host immune cells and protecting against antimicrobial peptides. We observed the presence of the *tox*A gene, which encodes for the toxin A protein, a known virulence factor in *B. pseudomallei*. Toxin A is involved in the disruption of host cell processes, particularly by targeting the host cell’s cytoskeleton, which impairs immune responses and promotes bacterial survival. Another key virulence factor identified was the type III secretion system (T3SS), which is associated with the ability to inject bacterial effector proteins directly into host cells, manipulating host cell functions to favor bacterial replication and immune evasion [44,45]

The isolate 584_2008/OEK contained *bla*OXA-57 and *bla*PenI β-lactamases, along with *amr*AB genes, encoding an efflux system. This is clinically relevant, as these genes may contribute to aminoglycoside resistance, a key factor in treatment failure. The outer membrane protein Omp38 was also detected, which is associated with low permeability and resistance to multiple antibiotics [61]. Omp38 is involved in the uptake of essential nutrients and plays a role in the resistance of the bacteria to host immune responses. In contrast, isolate 831_2019_NNK contained only the *bla*OXA-59 gene, which naturally occurs in *B. pseudomallei* strains, and is linked to resistance to β-lactam antibiotics. The main characteristics are summarized in Table 7.

Approximately 40% of the reported cases presented with respiratory or pulmonary infections, making this the most common clinical manifestation observed [35,67,70]. This finding aligns with previous studies indicating that pneumonia is one of the predominant forms of melioidosis. Most cases originated from Southeast Asia, particularly Thailand, reflecting the endemic nature of the disease in this region. The mean patient age was around 50 years, and the majority were male, consistent with the demographic profile typically described in the literature. Only one patient in the dataset was younger than 18 years old, representing less than 1% of all reported cases with a known age. This finding confirms that melioidosis predominantly affects adults, with pediatric cases being exceptionally rare among imported infections. The rarity of childhood cases may reflect both lower exposure risk and underdiagnosis in this age group [22]. These results highlight the importance of recognizing respiratory involvement in imported melioidosis and emphasize the need to consider travel history when evaluating patients with pneumonia of unclear origin. Only nine cases in the dataset involved central nervous system (CNS) manifestations, accounting for approximately 2–5% of all reports. These included meningoencephalitis, cerebral melioidosis, intracranial melioidosis, and brain abscesses—representing some of the most severe and uncommon forms of melioidosis. Notably, one additional case initially classified as a respiratory disease also exhibited brain abscesses, suggesting that CNS involvement may be underrecognized when it coexists with pulmonary infection. Clinically, these findings highlight the need for a high index of suspicion in returning travelers presenting with fever, headache, focal neurological deficits, or atypical pneumonia.

Early diagnosis and prolonged, targeted antimicrobial therapy are crucial to prevent fatal outcomes in neurological melioidosis. In addition, MRI findings can play a key role in supporting the diagnosis of neurological melioidosis. The presence of the so-called “tunnel sign”, reflecting the appearance of elongated, tubular abscess tracts within the brain parenchyma, has been described as a characteristic radiologic feature [78]. Recognition of this imaging pattern may assist in differentiating melioidosis from other pyogenic or granulomatous CNS infections and should prompt microbiological confirmation and appropriate antimicrobial therapy.

## 5. Conclusions

Melioidosis, often referred to as one of the “great mimickers,” presents a significant challenge for laboratories worldwide, especially in non-endemic areas. This is due to the absence of a pathognomonic clinical syndrome and the ability of *B. pseudomallei* to manifest clinical symptoms that mimic other diseases such as cancer, autoimmune disorders, and tuberculosis [9,32].

There is an urgent need to update diagnostic protocols for tropical or travel-related infections. We strongly recommend that all patients returning from endemic areas who present with fever be tested for melioidosis, in addition to the well-known “classic” tropical pathogens such as malaria, dengue, and rickettsial infections [35]. One of our goals is to raise awareness of potential *B. pseudomallei* infections in clinicians in Hungary, particularly among patients returning from endemic regions [16].

Today, next-generation sequencing technologies have become the most powerful and accurate tools for identifying and characterizing various pathogens. Epidemiological investigations should be extended to include geographic attribution and the determination of the origin of different infections, including RG3 pathogens [38,41]. Whole genome sequencing, combined with a robust bioinformatics pipeline, can aid in early diagnosis (e.g., *bim*A, *fha*B3, LPS, BTFC, and ITS identification) and provide insights into possible alternative treatments if necessary [44,45]. Furthermore, the ability of *B. pseudomallei* to form biofilms (which directly affects its reduced cultivation) and its prolonged survival in free-living organisms such as amoebas, along with its extreme adaptability, raises important biosecurity concerns. The potential for *B. pseudomallei* to adapt to temperate climates remains uncertain, due to the lack of comprehensive studies [5,13,43]. To address this phenomenon, further research is urgently needed.

## Figures and Tables

**Table 1:** *Burkholderia pseudomallei* strain collection in Hungary.

	Sample ID	Travel History	Incubation Period (Calculated from Arrival Home)	Specimen Type	Symptoms	Therapy	Antibiotic Therapy	Patient Status	Culture and Identification System
Case 1	584_OEK_2008	India	21 days	Blood	Weakness, fever, headache, facial neuralgia, sepsis	ICU supportive care + antibiotic therapy	Empiric therapy: Rocephin, Herpesin; after the diagnosis, meropenem 3 × 1 g/day and Amikacin 1 g/day for 18 days;	Recovered with long term neurological manifestations	Blood agar, chocolate agar followed by Biolog System
					CNS infection; CSF protein 1.69; Cell count 343; glucose 2.8; CRP elevated;				
Case 2	831_NNK_2019	Thailand	14 days	Abscess	Respiratory symptoms followed by neck pain, swollen lymph nodes	Ultrasound showed a neck abscess, which was surgically removed + antibiotic therapy	Amoxicillin Clavulanate followed the standard dosage	Fully recovered	Blood agar, chocolate agar followed by MALDI-TOF MS

CNS = central nervous system; CSF = cerebrospinal fluid; ICU = intensive care unit; CRP = C-reactive protein. Normal ranges: CSF protein 0.15–0.45 g/L; CSF glucose 2.5–4.4 mmol/L (≈45–80 mg/dL; ≈60–70% of concurrent blood g glucose); CSF white cell count, 0–5 cells/µL; serum CRP 0–5 mg/L.

**Table 2:** Main genome characteristics of the Hungarian strains.

	Isolate_1 (Signed 584_2008_OEK)	Isolate_2 (Signed 831_2019_NNK)
Genome size	7.2 Mb	7.13 Mb
GC content	67.9%	68.1%
CDS	5877	5857
Gene codes	7394	7460
Virulence-associated genes (annotated)	299	300
tRNAs	60	83
rRNAs	4	2
tmRNA	1	1
ncRNA	30	32
ncRNA regions	19	19
sORF	7	7
oriC	2	2
oriV	0	0
oriT	0	0
Gap	4	0
CRISPR-Cas systems	0	0

**Table 3:** Multilocus sequence typing results of the strains.

Strain ID	MLST									
	*ace*	*glt*B	*gmh*D	*lep*A	*lip*A	*nar*k	*ndh*	ST	Isolate ID	Genomic ID
584_2008_OEK	1	12	6	4	1	8	87	1643	5254	7517
831_2019_NNK	1	12	6	1	1	2	1	1051	6554	7518

**Table 4:** *Burkholderia pseudomallei* ST 1051 isolates from the PubMLST Database.

pubMLST ID	Isolate	Aliases	Country	Travel History	Year	Source
3230	BCC23	BPCC 24	Australia	na	1999	Environmental
3231	BCC24	BPCC 25	Australia	na	1999	Environmental
3232	BCC32	BPCC 33	Australia	na	2000	Environmental
5004	Ma154		India	no	2016	Human
5331	H 04-2015		Vietnam	no	2015	Human
5335	QB 03		Vietnam	no	2015	Human
5512	Ma-35		India	no	2012	Human
7518	831_2019_NNK		Hungary	Thailand	2019	Human

**Table 5:** Melioidosis cases in Europe between 1980 and 2025.

Country	Years	Number of Cases	Patient Characteristics	Source Country/Region	Clinical Presentation
United Kingdom	1988–2025	69	Male (n = 49), Female (n = 19); not specified (n = 1)	Thailand (n = 18); India (n = 3); Nigeria (n = 2); Banghladesh (n = 5); Cambogia (n = 2); Caribbean (n = 2); Ghana (n = 2); Malaysia (n = 2); Nigeria (n = 2); Borneo (n = 1); Singapore (n = 1); Vietnam (n = 1); China (n = 1); Shanghai (n = 1); Palau (n = 1); Pakistan (n = 1); Multiple country (n = 6), not specified (n = 6); Asia (n = 22)	Respiratory infection (n = 31), abscess (n = 13), sepsis (n = 17); genito-urinary infection (n = 6), gastrintestinal infection (n = 3), central nervous system infection (n = 2), cutaneus/soft tissue infection (n = 2);sore head (n = 1); not specified (n = 15)
The Netherlands	1990–2018	39	Male (n = 29), female (n = 10)	Thailand (n = 21), Brazil (n = 3); Vietnam (n = 3), Indonesia (n = 4); Gambia (n = 1); Sri Lanka (n = 1); Nepal (n = 1); Myanmar (n = 1); Malaysia (n = 1); Australia (n = 3), Panama (n = 1); Cambodia (n = 1); Australia (n = 1); not specified (n = 5),	Sepsis (n = 10), abscess (n = 10), respiratory infection (n = 20); genito-urinary infection (n = 6); central nervous system (n = 1), otitis externa (n = 1); mycotic aneurysm (n = 1)
Finland	1995–2014	4	Male (n = 3), female (n = 1)	Thailand (n = 4)	Cutaneous/soft tissues infection (n = 3), genito-urinary infection (n = 1)
Belgium	2001–2012	4	Male (n = 2), female (n = 2)	Bangladesh (n = 1); Vietnam (n = 1); Thailand (n = 1); Madagascar (n = 1)	Respiratory infection (n = 1), cutaneus/soft tissue infection (n = 1); genito-urinary infection (n = 1); lymphadenopathy (n = 1)
Denmark	1982–2024	9	Male (n = 6); Female (n = 1)	Kenya (n = 1); Thailand (n = 4); Laos (n = 1); Vietnam (n = 1); Cambodia (n = 1); Southeast Asia (n = 1)	Sepsis (n = 1); cutaneus/soft tissue infection (n = 1); genito-urinary infection (n = 1); abscess (n = 1); respiratory infection (n = 4)
Spain	2009, 2011, 2023–2024	5	Male (n = 4); Female (n = 1)	Gambia (n = 1); Guinea Bissau (n = 1); Senegal (n = 1); Madagascar (n = 1); West-Africa (n = 1); Colombia (n = 1); Thailand (n = 1)	Sepsis (n = 2); cutaneus/soft tissue infection (n = 1); abscess (n = 1); osteomyelitis (n = 1)
France	1995–2024	21	Male (n = 18), Female (n = 3)	Africa (n = 1); Thailand (n = 6); Cambodia (n = 4); Cameron (n = 1); Madagascar (n = 1); Guadalupe (n = 1), Vietnam (n = 3); Indonesia (n = 1); Phillippines (n = 1); not specified (n = 1)	Respiratory infection (n = 11); cervical lymphadenitis (n = 2); abdominal infection (n = 2); mycotic aneurysm (n = 3); saccular aneurysm (n = 1); central nervous system infection (n = 1); osteomyelytis (n = 1)
Germany	1996–2024	14	Male (n = 11); Female (n = 3)	South East Asia (n = 1); China (n = 1); Taiwan (n = 7); Maldives (n = 1); Sri Lanka (n = 1); Cambodia (n = 1); Thailand (n = 2), Vietnam (n = 1); Indonesia (n = 1); Mexico (n = 1); Dominican Republic (n = 1); USA (n = 1); Costa Rica (n = 1); No significant travel history (n = 1)	Respiratory infection (7); central nervous system infection (n = 1); abscess (n = 1); pericardial effusion (n = 1), urosepsis (n = 1); abdominal mycotic aortic aneurysm (n = 1); wound infection (n = 1)
Sweden	Not specified	5	Male (n = 4); Female (n = 1)	Thailand (n = 4); Malaysia (n = 1)	Respiratory infection (n = 1); central nervous system infection (n = 1); otitis externa (n = 1); abscess (n = 2); cutaneus/soft tissue (n = 1)
Austria	2014; 2020	2	Male (n = 2)	Thailand (n = 2)	Sepsis (n = 1); lymphadenitis (n = 1)
Hungary	2008–2019	2	Male (n = 1); Female (n = 1)	India, Thailand	Central nervous system infection (n = 1); abscess (n = 1)
Portugal	2011; 2025	3	Male (n = 1); Female (n = 1); Not specified (n = 1)	Thailand (n = 1), Brazil (n = 1), Not specified (n = 1)	Erythema nodosum (n = 1); sepsis (n = 1); gluteal abscess (n = 1); not specified (n = 1)
Slovenia	2007	1	Male (n = 1)	Asia (Brunei)	Osteomyelitis of parietal bone
Italy	1997, 2002, 2014,	3	Male (n = 2), female (n = 1)	Thailand (n = 2); Singapure (n = 1)	Respiratory infection (n = 3), multiple abscess (n = 1)
Norway	2011, 2014	3	Male (n = 3)	Sri Lanka (n = 1); Thailand (n = 1); Cambodia (n = 1)	Bacteraemia and splenic and prostatic abscesses (n = 2); neurological (n = 1)
Switzerland	2008–2012	4	Male (n = 4)	Thailand (n = 3), Caribbean, Martinique Island (n = 1)	Respiratory infection (n = 1); abscess (n = 1); systemic inflammatory response syndrome (n = 1); cutaneous/soft tissues (n = 1)
Iceland	Not specified	4	Male (n = 4)	Thailand (n = 3); Southeast Asia (n = 1); not specified (n = 1)	Respiratory infection (n = 2); necrotizing granulomatous inflammation (n = 1); pleural abscess (n = 1); osteomyelitis (n = 1)

**Table 6:** Neuromelioidosis cases in Europe.

Country	Year	Travel Anamnesis	Clinical Picture	Reference
The Netherlands	1993	Not specified	Meningoencephalitis	[67]
Hungary	2008	India	Meningoencephalitis	in this study
Norway	2014	Cambogia	Cerebral abscess	[71]
The Netherlands	2015	Thailand	Brain abscesses	[67]
Germany	2018	Thailand	Cerebral melioidosis	[72]
Sweden	2021	Thailand	Intracranial melioidosis	[73]
Germany	2022	Unknown, several countries, possibility of autochthonous infection	Neuromelioidosis	[29]
France	2023	Thailand	Pneumonia, Meningitis	[74]
United Kingdom	2024	Sri Lanka	Neuromelioidosis	[35,69]

**Table 7:** Main genomic and phenotypic characteristics of the Hungarian *B. pseudomallei* isolates.

Feature	Isolate 584_2008_OEK	Isolate 831_2019_NNK	Implications
Clinical manifestation	Neuromelioidosis (meningoencephalitis)	Neck abscess	Bm variant of *bimA*, associated with neurological disease
MLST/sequence type (ST)	ST 1640 (novel)	ST 1051	ST 1051 shows overlap with Australian strains
*bim*A variant	Bm	Bp	Bm variant linked to neurological complications
YLF gene cluster	Present	Present	Enhances adhesion and biofilm formation; common in Asian strains
BTFC cluster	Absent	Absent	Characteristic of Australian isolates; affects motility/chemotaxis
ITS type	Type C (622 bp)	Type C (622 bp)	Commonly observed in Asian isolates
*fha*B3	Present	Present	Adhesion and tissue invasion; contributes to virulence
Capsule polysaccharide operon (*wcb*)	Present, no key mutations	Present, no key mutations	Important for immune evasion
Toxin A (*tox*A)	Present	Present	Disrupts host cell cytoskeleton
T3SS	Present	Present	Type III secretion system; facilitates host cell manipulation
β-lactamase genes	*bla*OXA-57, *bla*PenI, *amr*AB	*bla*OXA-59	Contributes to antibiotic resistance (aminoglycosides, β-lactams)
Geographical link/phylogeny	First neuromelioidosis case in Europe	Linked to Asian strains via YLF cluster	Highlights travel-related risk and global strain diversity

## Data Availability

Sequences have been deposited at the GenBank under the accession of SUB12019197, BioSample: SAMN26814095 and SAMN26814096.

## Supplementary Materials

The following supporting information can be downloaded at: https://www.mdpi.com/article/10.3390/pathogens14111108/s1. Supplementary Data S1: *Burkholderia pseudomallei* isolates used in this study to perform cgMLST. Supplementary Data S2: Travel related melioidosis cases in Europe between 1980–2025. Supplementary Data S3: Presence of the main *Burkholderia*-related virulence genes according to VFDB. Supplementary Data S4: Presents the different types of virulence genes between the two isolates.

## Abbreviations

The following abbreviations are used in this manuscript:

ABC	ATP-binding cassette transporters
AraC	Transcriptional regulator protein
ATP	Adenosine triphosphate
BapA	Bacterial adhesion protein
BCC	Burkholderia cepacia complex
BPC	*Burkholderia pseudomallei* complex
BimA	Bacterial invasion protein
BipB, BipC, BipD	Bip proteins are part of the type III secretion system (T3SS)
BopA	Bacterial outer membrane protein involved in adhesion or invasion mechanisms
cgMLST	Core genome multilocus sequence typing
CRISPRs	Clustered regularly interspaced short palindromic repeats
CheA, CheD, CheW, CheY	Proteins involved in chemotaxis
DNA	Deoxyribonucleic acid
ITS	Internal transcribed spacer
LPS	Lipopolysaccharide
MALDI-TOF	Matrix-assisted laser desorption/ionization time of flight
MLST	Multilocus sequence typing
OMP	Outer membrane proteins
PCR	Polymerase chain reaction
qPCR	Quantitative PCR
RNA	Ribonucleic acid
SNP	Single nucleotide polymorphism
T3SS	Type III secretion system
T6SS1	Type 6 secretion system- cluster 1
VF	Virulence factors
VFDB	Virulence factor database
WGS	Whole genome sequencing
YadA	Bacterial adhesion protein
