# Supplementary material for: Whole Genome Sequencing and Comparative Genomics of the Emerging Pathogen Burkholderia pseudomallei Isolated from Two Travel-Related Infections in Hungary
Source: Pathogens. 2025 Oct 31;14(11):1108. doi: 10.3390/pathogens14111108 (PMC12654974; doi:10.3390/pathogens14111108)
Supplement: Supplementary file 1 [file pathogens-14-01108-s001.zip › Supplementary Data 3.pdf]

Supplementary Data 3. Presence of the main *Burkholderia*-related virulence genes according to VFDB.

**VFDB ID**	**Product**	**Target gene**	**584_OEK_2008**	**831_NNK_2019**
VFG002545	acetyltransferase	BPSL2786	yes	yes
VFG002446	Type III secretion system secreted protein BapA	*bap*A	yes	no
VFG002445	acyl carrier protein	*bap*B	yes	yes
VFG002444	invasion protein	*bap*C	yes	yes
VFG002468	Type III secretion system protein BsaJ	*bas*J	yes	yes
VFG002451	chaperone	*bic*A	yes	yes
VFG002441	chaperone	*bic*P	yes	yes
VFG002424	YadA family of bacterial autotransporter mediating actin tail formation	*bim*A	yes	yes
VFG002450	Type III secretion system translocator protein BipB	*bip*B	yes	yes
VFG002449	Type III secretion system translocator protein BipC	*bip*C	yes	yes
VFG002447	Type III secretion system needle tip protein BipD	*bip*D	yes	yes
VFG002436	surface-exposed protein_ autotransporter	*boa*A	yes	yes
VFG002442	putative Type III secretion system effector BopA	*bop*A	yes	yes
VFG002473	Type III secretion system effector BopC	*bop*C	yes	yes
VFG002443	Type III secretion system secreted effector_ G-nucleotide exchange factor	*bop*E	yes	yes
VFG002448	HNS-like regulatory protein	*bpr*A	yes	yes
VFG002440	two-component response regulator	*bpr*B	yes	yes
VFG002438	AraC family transcriptional regulator_ regulates basal T6SS1 expression	*bpr*C	yes	yes
VFG002439	Type III secretion system secreted protein BprD	*bpr*D	yes	yes
VFG002471	regulatory protein	*bpr*P	yes	yes
VFG002467	Type III secretion system protein BsaK	*bsa*K	yes	yes
VFG002466	Type III secretion system protein BsaL	*bsa*L	yes	yes
VFG002465	Type III secretion system protein BsaM	*bsa*M	yes	yes
VFG002464	AraC family transcriptional regulator_ regulates basal T6SS1 expression	*bsa*N	yes	yes
VFG002463	Type III secretion system protein BsaO	*bsa*O	yes	yes
VFG002462	Type III secretion system gate keeper protein	*bsa*P	yes	yes
VFG002461	Type III secretion system protein BsaQ	*bsa*Q	yes	yes

VFG002460	Type III secretion system protein BsaR	*bsa*R	yes	yes
VFG002459	ATP synthase	*bsa*S	yes	yes
VFG002458	Type III secretion system protein BsaT	*bsa*T	yes	yes
VFG002456	Type III secretion system protein BsaV	*bsa*V	no	yes
VFG002457	Type III secretion system protein BsaU	*bsa*U	yes	no
VFG002456	Type III secretion system protein BsaV	*bsa*V	yes	no
VFG002454	Type III secretion system protein BsaX	*bsa*X	yes	yes
VFG002453	Type III secretion system protein BsaY	*bsa*Y	yes	yes
VFG002452	YscU homolog_ component of the inner membrane ring	*bsa*Z	yes	yes
VFG002539	N-acylhomoserine lactone synthase	*bsp*I2	yes	yes
VFG002541	N-acylhomoserine lactone synthase	*bsp*I3	yes	yes
VFG002540	N-acyl-homoserine lactone dependent regulatory protein	*bsp*R2	yes	yes
VFG002542	N-acylhomoserine lactone-dependent regulatory protein	*bsp*R3	yes	yes
VFG002543	LuxR family transcriptional regulator	*bsp*R4	yes	yes
VFG045467	cyclic di-GMP phosphodiesterase	*cdp*A	yes	yes
VFG041780	Type III secretion system secreted effector CHBP_ cyclomodulin Cif homolg	*chb*p	yes	yes
VFG002531	chemotaxis two-component sensor kinase CheA	*che*A	yes	yes
VFG002526	chemotaxis-specific methylesterase	*che*B	yes	yes
VFG002527	chemoreceptor glutamine deamidase CheD	*che*D	yes	yes
VFG002528	chemotaxis protein methyltransferase	*che*R	yes	yes
VFG002530	chemotaxis protein CheW	*che*W	yes	yes
VFG002525	chemotaxis protein CheY	*che*Y	yes	yes
VFG002532	chemotaxis two-component response regulator CheY1	*che*Y1	yes	yes
VFG002524	chemotaxis regulator CheZ	*che*Z	yes	yes
VFG002507	flagellar basal body P-ring biosynthesis protein FlgA	*flg*A	yes	yes
VFG002508	flagellar basal-body rod protein FlgB	*flg*B	yes	yes
VFG002509	flagellar basal body rod protein FlgC	*flg*C	yes	yes
VFG002510	flagellar basal-body rod modification protein FlgD	*flg*D	yes	yes
VFG002511	flagellar hook protein FlgE	*flg*E	yes	yes

VFG0025 12	flagellar basal body rod protein FlgF	*flg*F	yes	yes
VFG0025 13	flagellar basal body rod protein FlgG	*flg*G	yes	yes
VFG0025 14	flagellar L-ring protein precursor FlgH	*flg*H	yes	yes
VFG0025 15	flagellar P-ring protein precursor FlgI	*flg*I	yes	yes
VFG0025 16	flagellar rod assembly protein/muramidase FlgJ	*flg*J	yes	yes
VFG0025 17	flagellar hook-associated protein 1 FlgK	*flg*K	yes	yes
VFG0025 18	flagellar hook-associated protein 3 FlgL	*flg*L	yes	yes
VFG0025 06	negative regulator of flagellin synthesis	*flg*M	yes	yes
VFG0025 05	flagella synthesis protein FlgN	*flg*N	yes	yes
VFG0025 22	flagellar biosynthesis protein FlhA	*flh*A	yes	yes
VFG0025 23	flagellar biosynthesis protein FlhB	*flh*B	yes	yes
VFG0025 21	flagellar biosynthesis regulator FlhF	*flh*F	yes	yes
VFG0025 20	flagellar biosynthesis protein FlhG	*flh*G	yes	yes
VFG0025 19	flagellar biosynthesis sigma factor	*fli*A	yes	yes
VFG0025 35	flagellin	*fli*C	yes	yes
VFG0025 36	flagellar capping protein FliD	*fli*D	yes	yes
VFG0025 03	flagellar hook-basal body complex protein FliE	*fli*E	yes	yes
VFG0025 02	flagellar M-ring protein FliF	*fli*F	yes	yes
VFG0025 01	flagellar motor switch protein G	*fli*G	yes	yes
VFG0025 00	flagellar assembly protein H	*fli*H	yes	yes
VFG0024 99	flagellum-specific ATP synthase FliI	*fli*I	yes	yes
VFG0024 98	flagellar protein FliJ	*fli*J	yes	yes
VFG0024 97	flagellar hook-length control protein FliK	*fli*K	yes	yes
VFG0024 90	flagellar basal body protein FliL	*fli*L	yes	yes
VFG0024 91	flagellar motor switch protein FliM	*fli*M	yes	yes
VFG0024 92	flagellar motor switch protein FliN	*fli*N	yes	yes
VFG0024 93	flagellar protein FliO	*fli*O	yes	yes
VFG0024 94	flagellar biosynthesis protein FliP	*fli*P	yes	yes
VFG0024 95	flagellar biosynthesis protein FliQ	*fli*Q	yes	yes

VFG0024 96	flagellar biosynthetic protein FliR	*fli*R	yes	yes
VFG0025 04	flagellar protein FliS	*fli*S	yes	yes
VFG0025 54	phosphoheptose isomerase	*gmh*A	yes	yes
VFG0025 69	GDP-mannose pyrophosphorylase	*man*C	yes	yes
VFG0025 34	flagellar motor protein MotA	*mot*A	yes	yes
VFG0025 33	flagellar motor protein MotB	*mot*B	yes	yes
VFG0024 69	Type III secretion system protein	*org*A	yes	yes
VFG0024 70	Type III secretion system protein	*org*B	yes	yes
VFG0024 25	type IV fimbrial pilin protein	*pil*A	yes	yes
VFG0024 26	type IV pilus assembly protein	*pil*B	yes	yes
VFG0024 27	type IV pilus assembly protein PilC	*pil*C	yes	yes
VFG0024 28	type IV prepilin leader peptide type M1	*pil*D	yes	yes
VFG0429 66	type IV pilus biosynthesis protein PilL	*pi*L	yes	yes
VFG0429 64	type IV pilus inner membrane platform protein PilM	*pil*M	yes	yes
VFG0024 34	type IV pilus inner membrane platform protein PilN	*pil*N	yes	yes
VFG0024 33	type IV pilus inner membrane platform protein PilO	*pil*O	yes	yes
VFG0429 65	type IV pilus biogenesis protein PilP	*pil*P	yes	yes
VFG0024 32	type IV pilus biosynthesis protein	*pil*Q	yes	yes
VFG0024 31	type IV pilus biosynthesis protein PilR	*pil*R	yes	yes
VFG0024 30	major pilin subunit	*pil*S	yes	yes
VFG0024 35	twitching motility protein	*pil*T	yes	yes
VFG0024 29	type IV pilus biogenesis protein PilV	*pil*V	yes	yes
VFG0025 37	N-acylhomoserine lactone synthase	*pml*I *bsp*I1	yes	yes
VFG0025 38	N-acylhomoserine lactone dependent regulatory protein	*pml*R *bsp*R1	yes	yes
VFG0024 55	surface presentation of antigens protein SpaP	*spa*P	yes	yes
VFG0024 82	Type VI secretion system protein	*tag*AB-5	yes	yes
VFG0024 83	Type VI secretion system protein	*tag*B-5	yes	yes
VFG0024 84	Type VI secretion system protein TssF	*tag*C-5	yes	yes
VFG0024 85	Type VI secretion system protein TssG	*tag*D-5	yes	yes

VFG0025 29	methyl-accepting chemotaxis protein I	*tsr*	yes	yes
VFG0024 74	Type VI secretion system protein TssA	*tss*B-5	yes	yes
VFG0024 75	Type VI secretion system protein TssB	*tss*C-5	yes	yes
VFG0024 76	hemolysin-coregulated protein Hcp1	*tss*D-5	yes	yes
VFG0024 77	Type VI secretion system protein TssC	*tss*E-5	yes	yes
VFG0024 78	Type VI secretion system protein TssD	*tss*F-5	yes	yes
VFG0024 79	Type VI secretion system protein TssE	*tss*G-5	yes	yes
VFG0024 80	Clp-type ATPase chaperone protein	*tss*H-5 *clp*V	yes	yes
VFG0024 81	Type VI secretion system protein VgrG1	*tss*I-5	yes	yes
VFG0024 86	Type VI secretion system protein TssH	*tss*J-5	yes	yes
VFG0024 87	Type VI secretion system protein TssI	*tss*K-5	yes	yes
VFG0024 88	Type VI secretion system protein TssJ	*tss*L-5	yes	yes
VFG0024 89	icmF-like protein	*tss*M-5	yes	yes
VFG0025 68	capsule polysaccharide export protein	*wcb*A	yes	yes
VFG0025 67	capsular polysaccharide glycosyltransferase biosynthesis protein	*wcb*B	yes	yes
VFG0025 66	capsular polysaccharide biosynthesis/export protein	*wcb*C	yes	yes
VFG0025 65	capsule polysaccharide export ABC transporter transmembrane protein	*wcb*D	yes	yes
VFG0025 62	glycosyltransferase	*wcb*E	yes	yes
VFG0025 61	capsule polysaccharide biosynthesis protein	*wcb*F	yes	yes
VFG0025 60	capsular polysaccharide biosynthesis protein	*wcb*G	yes	yes
VFG0025 59	glycosyl transferase	*wcb*H	yes	yes
VFG0025 58	capsular polysaccharide biosynthesis protein	*wcb*I	yes	yes
VFG0025 57	capsular polysaccharide biosynthesis protein	*wcb*J	yes	yes
VFG0025 56	GDP sugar epimerase/dehydratase	*wcb*K	yes	yes
VFG0025 55	sugar kinase	*wcb*L	yes	yes
VFG0025 53	D-glycero-d-manno-heptose 1-phosphate guanosyltransferase	wcbM	yes	yes
VFG0025 52	D-glycero-d-manno-heptose 1_7-bisphosphate phosphatase	*wcb*N	yes	yes
VFG0025 51	capsule polysaccharide biosynthesis/export protein	*wcb*O	yes	yes
VFG0025 50	capsular polysaccharide biosynthesis dehydrogenase/reductase	*wcb*P	yes	yes

VFG0025 49	capsular polysaccharide biosynthesis transmembrane protein	*wcb*Q	yes	yes
VFG0025 48	capsular polysaccharide biosynthesis fatty acid synthase	*wcb*R	yes	yes
VFG0025 47	UDP-3-O-[3-hydroxymyristoyl] N-acetylglucosamine deacetylase	*wcb*S	yes	yes
VFG0025 46	acyl-CoA transferase	*wcb*T	yes	yes
VFG0025 64	capsular polysaccharide export ABC transporter transmembrane protein	*wzm*	yes	yes
VFG0025 63	ATP-binding ABC transporter capsular polysaccharide export protein	*wzt*2	yes	yes
