# Supplementary material for: Whole Genome Sequencing and Comparative Genomics of the Emerging Pathogen Burkholderia pseudomallei Isolated from Two Travel-Related Infections in Hungary
Source: Pathogens. 2025 Oct 31;14(11):1108. doi: 10.3390/pathogens14111108 (PMC12654974; doi:10.3390/pathogens14111108)
Supplement: Supplementary file 1 [file pathogens-14-01108-s001.zip › Supplementary Data 4.pdf]

Supplementary Data 4. Presents the different types of virulence genes between the two isolates.

	584_OEK_2008	831_NNK_2019
Protein Metabolism	313	220
Miscellaneous	102	48
Cell Division and Cell Cycle	41	0
Sulfur Metabolism	87	33
Dormancy and Sporulation	2	1
Secondary Metabolism	7	8
Cell Wall and Capsule	233	54
Virulence, Disease and Defense	143	81
Fatty Acids, Lipids, and Isoprenoids	205	88
Membrane Transport	339	168
Regulation and Cell signaling	103	38
Photosynthesis	0	0
Stress Response	179	94
Respiration	181	139
DNA Metabolism	110	66
Metabolism of Aromatic Compounds	156	80
Phosphorus Metabolism	63	31
Cofactors, Vitamins, Prosthetic Groups, Pigments	372	229
Carbohydrates	527	300
Potassium metabolism	14	8
RNA Metabolism	214	57
Motility and Chemotaxis	87	18
Amino Acids and Derivatives	746	500
Nucleosides and Nucleotides	123	94
Nitrogen Metabolism	108	65
Phages, Prophages, Transposable elements, Plasmids	5	6
Iron acquisition and metabolism	58	65
